# Pediatric eosinophilic esophagitis among children attending a tertiary hospital in Nairobi, Kenya: a case series

**DOI:** 10.3389/falgy.2026.1728648

**Published:** 2026-07-21

**Authors:** Lynnette Mwangi, Hassan Silim, Faith Chebet, Utpol Chowdhury, Waceke Kombe

**Affiliations:** 1 Department of Paediatrics, The Aga Khan University Hospital, Nairobi, Kenya; 2 Department of Paediatrics, The Aga Khan Hospital, Mombasa, Kenya

**Keywords:** case series, dysphagia, eosinophilic esophagitis, Kenya, pediatrics

## Abstract

**Background:**

Eosinophilic esophagitis (EoE) is an emerging chronic immune-mediated esophageal disorder characterized by esophageal dysfunction and dense eosinophilic infiltration of the epithelium. Despite increasing global recognition, data from sub-Saharan Africa remain scarce.

**Objective:**

The aim of this work is to describe the clinical spectrum, endoscopic and histopathologic features, and treatment outcomes of pediatric EoE cases diagnosed at a tertiary private hospital in Nairobi, Kenya.

**Methods:**

We conducted a retrospective case series of eight children, aged 4–17 years, who were diagnosed with EoE at The Aga Khan University Hospital, Nairobi. Data collected included demographics, presenting symptoms, laboratory findings, endoscopic features, histology, treatment regimens, and follow-up outcomes. Diagnosis was based on the presence of ≥15 eosinophils per high-power field in esophageal biopsies, alongside clinical and endoscopic correlation, and exclusion of other causes of esophageal eosinophilia.

**Results:**

Of the eight patients (six boys, two girls), common symptoms included dysphagia (*n* = 2, 25%), chronic abdominal pain (*n* = 5, 62%), and globus sensation (*n* = 1, 12%). Endoscopic findings included edema, furrows, exudates, and white plaques, classified using the Eosinophilic Esophagitis Endoscopic Reference Score (EREFS). Eosinophil counts ranged from 10 to 40 eosinophils per high-power field, demonstrating variability in symptom presentation and endoscopic findings. Two patients with eosinophil counts <15/hpf were classified as suspected EoE and treated empirically. Five patients had a history of atopy, and two had positive food allergy panels for wheat and dairy. Treatment with budesonide slurry and dietary elimination of wheat and dairy achieved clinical remission in six of eight cases. Follow-up endoscopy and histopathological assessment were available for three patients, all of whom achieved histological remission, defined as the resolution of esophageal eosinophilia on repeat biopsy. No severe adverse events were reported.

**Conclusion:**

This case series highlights EoE as an important yet often overlooked cause of upper gastrointestinal symptoms in Kenyan children. Its ability to mimic other gastrointestinal disorders warrants heightened clinical suspicion, particularly in cases of refractory gastritis or dysphagia unresponsive to proton pump inhibitors. Effective management includes dietary elimination and topical corticosteroids. Larger multicenter studies are needed to define the epidemiology, diagnostic challenges, and long-term outcomes of pediatric EoE in sub-Saharan Africa and to inform clinical guidelines.

## Introduction

Eosinophilic esophagitis (EoE) is a chronic esophageal inflammatory disorder marked by esophageal dysfunction and elevated eosinophil counts in the epithelium. Diagnosis requires recurrent gastrointestinal symptoms, increased eosinophils per high-power field (HPF) upon microscopic assessment of a biopsy, and the exclusion of other eosinophilic gastrointestinal disorders, such as eosinophilic gastritis, eosinophilic enteritis, or eosinophilic colitis. Although the exact cause of EoE remains unclear, immune-mediated responses to food antigens, such as soy, nuts, cow's milk, eggs, or shellfish, are key triggers. The majority of patients have a history of atopy and improve with the application of topical steroids and the removal of dietary antigens (1, 2). Genetic predisposition also plays a role, with four genetic loci [5q22 (TSLP/WDR36), 2p23 (CAPN14), 11q13 (LRRC32/EMSY), and 16p13 (CLEC16A/DEX1)] with genome-wide significance identified across multiple studies (3–7).

The pediatric incidence and prevalence are estimated at 5.1 cases/year and 19.1 cases per 100,000 children (8). However, local data are scarce, highlighting the need for epidemiological studies in this region. This case series aims to describe the clinical presentation, diagnostic approaches, management, and short-term outcomes of pediatric EoE in Nairobi, Kenya. This research is important because socioeconomic background, dietary patterns, and environmental factors play a significant role in disease prevalence differences. Furthermore, the scarcity of specialized pediatric gastroenterology services in the region makes diagnosis and early referral to gastroenterologists a significant challenge (9).

## Methodology

We conducted a retrospective case series of consecutive pediatric patients diagnosed with EoE at the pediatric gastroenterology clinic of the Aga Khan University Hospital in Nairobi, Kenya, between April 2024 and April 2025. This institution is a Level 6 private, tertiary, teaching, and referral hospital. The pediatric gastroenterology clinic provides comprehensive, specialized care for children with gastrointestinal, liver, and nutritional disorders, including diagnostic endoscopy, multidisciplinary management, and follow-up of chronic conditions.

Cases were identified retrospectively through a review of pediatric gastroenterology clinic records, endoscopy logs, and histopathology reports. Identification of cases depended on clinical recognition and documentation by the attending pediatric gastroenterologist during routine clinical care, along with histopathologic confirmation from esophageal biopsies. Diagnosis was established based on compatible clinical features and histopathological confirmation from esophageal mucosal biopsies, with a peak eosinophil density of ≥15 eosinophils per high-power field, in accordance with ESPGHAN (European Society for Paediatric Gastroenterology, Hepatology and Nutrition) criteria.

Parasitic, fungal, and infectious esophagitis were excluded based on stool analysis and biopsy reports. Patients demonstrated no eosinophilia in other sections of the gastrointestinal tract, as per biopsy findings. Proton pump inhibitor (PPI)-responsive esophageal eosinophilia was treated as a subset of EoE and was the treatment modality in some of the cases described. Gastroesophageal reflux disease (GERD) was ruled out empirically, as all patients had initially been treated with proton pump inhibitors but remained refractory despite adequate use.

Verbal consent was obtained via telephone from parents/guardians, and the conversations were audio-recorded with consent and appropriately documented. Patient data were categorized into the following age groups: 4–8, 9–13, and 14–18 years. Repeat endoscopy and histopathological reassessment were not uniformly performed in all patients and were guided by clinical response and the treating physician's discretion, rather than a standardized study protocol.

Given the retrospective nature of the study and the reliance on gastroenterologist recognition of the cases, there is potential for selection bias and incomplete case capture. Children with mild, atypical, or non-specific symptoms may not have been referred for a gastroenterology review or undergone an endoscopy and biopsy, resulting in possible underdiagnoses. Similarly, patients managed empirically for refractory gastritis, gastroesophageal reflux disease, constipation, or functional abdominal pain without an esophageal biopsy may have been overlooked. Consequently, this series may preferentially represent patients with more severe, persistent, or clinically recognized disease presenting at a tertiary referral center, thereby limiting the generalizability of the findings.

### Case report 1

#### Presenting symptoms

An adolescent boy in the age bracket of 9–13 years presented with a 4-year history of intermittent epigastric and periumbilical pain (severity 8/10). The pain was described as burning and non-radiating, and exacerbated by oily, spicy foods and wheat. Associated symptoms included nausea, non-bilious vomiting, a globus sensation, and intermittent dysphagia for solids. Initial management with esomeprazole, alginate, sucralfate, and a wheat-free diet provided only partial relief.

Physical examination revealed a body mass index (BMI) of 15.5 (weight 49 kg and height 1.78 m) and mild epigastric tenderness, with the rest of the examination being unremarkable.

#### Past medical history

The patient had a history of GERD in infancy that was treated with esomeprazole, with resolution by 6 months of age. His bowel habits were notable for intermittent constipation with the passage of hard, pellet-like stools (Bristol stool chart type 1), associated with abdominal fullness. There was no history of straining when passing stool, fecal incontinence, or hematochezia. He experienced loose stools after ingesting wheat, but not after consuming dairy. Additionally, he had had lower-limb skin lesions since childhood, evolving from papules and macules to residual scarring. Similar lesions were reported in his siblings.

He also reported bilateral morning joint pain affecting his fingers and elbows, accompanied by stiffness that improved during the day. There was no associated swelling, color change, or temperature variation in the joints. His medical history included self-resolving oral ulcers and occasional photic eye pain without visual symptoms. There was no history of fever, jaundice, weight loss, or gastrointestinal bleeding.

The patient had experienced seven previous admissions due to gastritis and constipation, along with frequent outpatient visits for gastritis. He was born at term following an uneventful delivery. He had no developmental delays and family history is unremarkable.

#### Endoscopic findings

**Figure 1:**
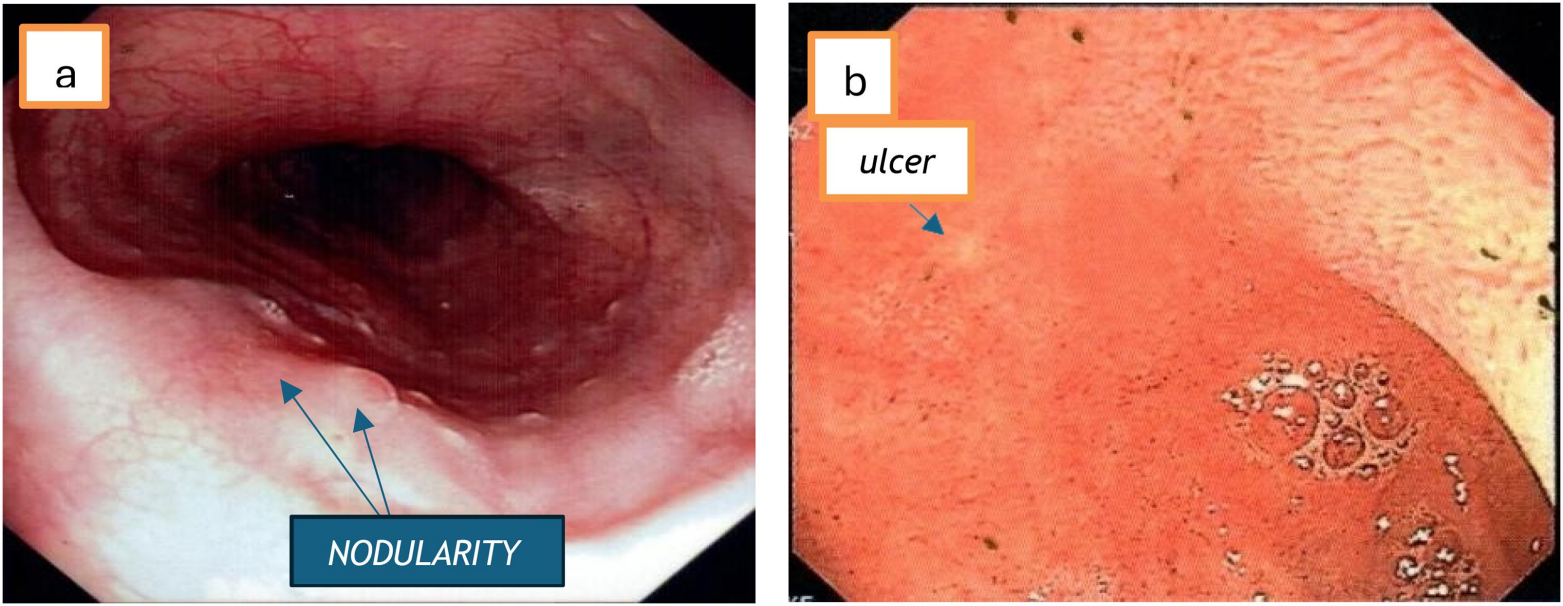
**(a)** Esophageal nodularity and **(b)** duodenal ulcer for case 1.

Upper endoscopy revealed severe distal esophageal nodularity and a duodenal ulcer-like lesion. No furrows, rings, edema, or exudates were seen [Eosinophilic Esophagitis Endoscopic Reference Score (EREFS) 1] (Figure 1).

*Distal esophagus:* moderate chronic esophagitis with >40 eosinophils/HPF in the lamina propria, without spongiosis or basal zone hyperplasia.
*Gastric antrum*: mild active chronic inflammation with 2–8 eosinophils/HPF.
*Gastric body*: mild chronic inflammation with 0–2 eosinophils/HPF.

#### Other investigations

Laboratory tests revealed leukopenia (lowest WBC count 3.31 × 10^9^/L) with neutropenia and a normal eosinophil count. Immunoglobulin levels, along with T- and B-cell flow cytometry, were normal. Thyroid profile, tissue transglutaminase IgA, liver function tests, amylase, and lipase were normal. Initial stool analysis detected *Giardia lamblia* and *Blastocystis hominis*, both of which were subsequently treated. Stool antigen testing for *Helicobacter pylori* was positive twice but negative after triple therapy. An abdominal X-ray showed pancolonic fecal loading, while an abdominal ultrasound was normal.

#### Treatment approach

The patient was diagnosed with confirmed EoE. He began a 5-day course of prednisolone at 20 mg daily, followed by budesonide slurry at a dosage of 2 mg twice daily for 10 weeks. The slurry was reconstituted by mixing the budesonide respules with honey. During that time, he followed a wheat- and dairy-free diet. He reported marked improvement during treatment, with successful gradual reintroduction of wheat-based products.

#### Follow-up and outcome

A repeat esophagogastroduodenoscopy (OGD) performed 26 days after completing the budesonide course demonstrated histologic remission. Eosinophil counts decreased to 0 eosinophils/HPF in the mid-esophagus and 0–1 eosinophils/HPF in the distal esophagus. However, chronic gastritis was noted alongside a positive test for *H. pylori*. He was treated for the infection, and a gastric biopsy was conducted for *H. pylori* culture, which showed no growth.

### Case report 2

#### Presenting symptoms

An adolescent boy in the age bracket of 14–18 years, with a history of asthma and migraines, presented with a 2-year history of intermittent epigastric pain, nausea, and vomiting. He also had chronic constipation that required fecal disimpaction and ongoing polyethylene glycol therapy. He denied dysphagia, odynophagia, food impaction, or the need for excessive oral fluids. Imaging suggested a jejunal duplication cyst, but he was ultimately diagnosed with EoE.

#### Past medical history

The patient had a history of asthma since the age of 6 and migraines for the previous 4 years. He had been managed at a pediatric gastroenterology clinic for chronic constipation. He required fecal disimpaction and was maintained on polyethylene glycol (3350), which he continues to use.

#### Endoscopic findings

**Figure 2:**
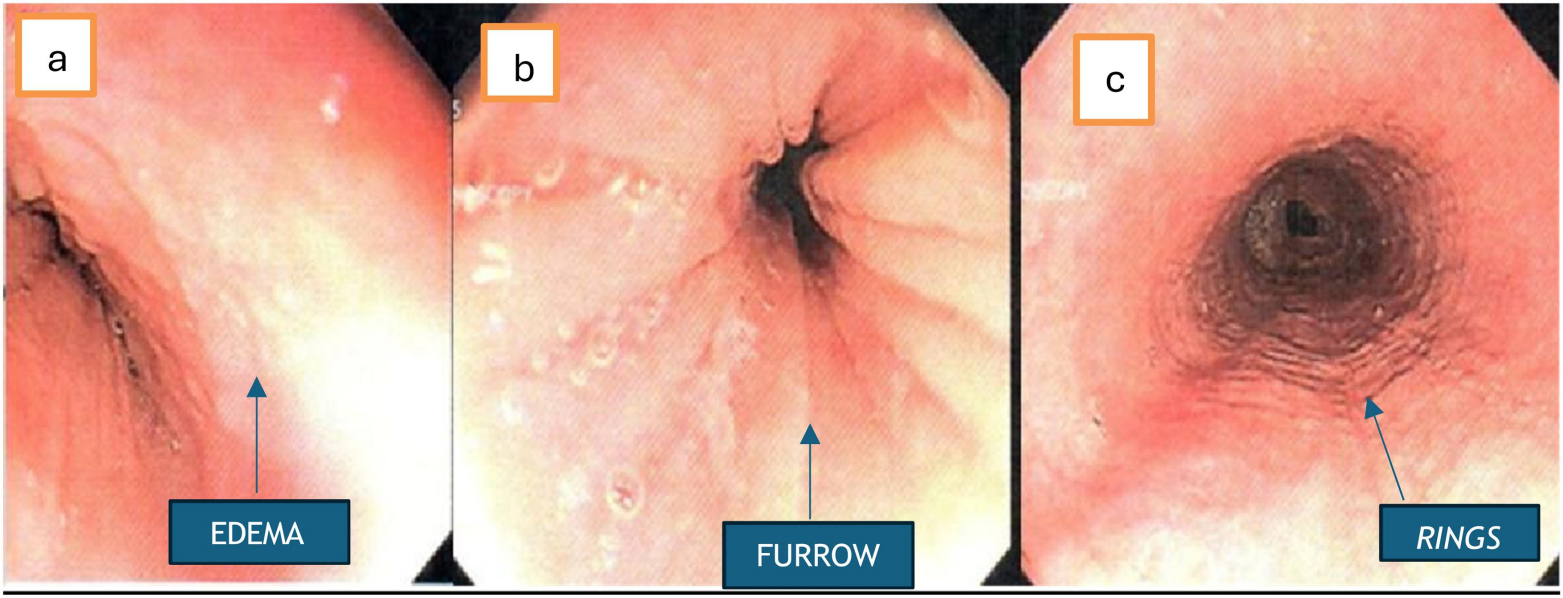
**(a)** Edema, **(b)** furrows, and **(c)** rings for case 2.

Upper endoscopy demonstrated edema, exudates, rings, and furrows in the mid and lower esophagus, with an EREFS of 4. Colonoscopy was normal (Figure 2).

*Distal esophagus:* esophagitis with spongiosis and basal zone hyperplasia, with 20–22 eosinophils/HPF.
*Gastric antrum and body:* chronic gastritis with 2–3 eosinophils/HPF and negative *H. pylori* test.
*Colon:* normal.

#### Other investigations

The patient's renal function and liver panel were normal. The peripheral eosinophil percentage was elevated, but the absolute eosinophil count remained within normal limits. Fecal calprotectin levels were normal. Serum-specific IgE testing for food allergies indicated hypersensitivity to egg white, fish, wheat, peanut, and soybean.

An abdominal computed tomography (CT) showed a suspected enteric duplication cyst, measuring approximately 6.4 cc, located in the left upper quadrant of the proximal jejunum. He was managed conservatively with esomeprazole and polyethylene glycol and allowed to go home. However, the patient returned presenting with severe left upper quadrant abdominal pain associated with vomiting but remained apyrexial. In view of these acute symptoms and CT findings, he underwent exploratory laparotomy. No duplication cysts were found, but the duodenal-jejunal flexure was deflected to the right. The duodenal-jejunal flexure was dissected and freed from the surrounding tissue, with the remainder of the bowel appearing normal.

#### Treatment approach

Following histological confirmation of EoE, the patient commenced a six-food elimination diet (CFED) with nutritional support. He continued esomeprazole and polyethylene glycol. Topical budesonide was deferred due to the recent laparotomy to allow for wound healing.

#### Follow-up and outcome

The patient remains under follow-up in the pediatric gastroenterology clinic and has shown clinical improvement in gastrointestinal symptoms. A repeat esophagogastroduodenoscopy with biopsy has not yet been performed; therefore, histologic remission could not be assessed at the time of writing this article.

### Case report 3

#### Presenting symptoms

A girl in the age bracket of 4–8 years presented with a 2-year history of progressive feeding difficulty, preferring semisolid and liquid foods, with prolonged feeding times exceeding 2 h. She also experienced intermittent central chest and periumbilical abdominal pain, along with subjective weight loss.

On examination, her weight and height were at the 72nd and 75th percentiles, respectively, and she had mild epigastric tenderness.

#### Past medical history

Previously, the patient had been treated for constipation, which resolved. She also had a history of chronic rhinosinusitis, adenoid hypertrophy (treated surgically), and prematurity at 30 weeks due to maternal pre-eclampsia, requiring 2 months in the neonatal intensive care unit. She also received treatment for retinopathy of prematurity. Her growth and development were otherwise appropriate.

#### Endoscopic findings

**Figure 3:**
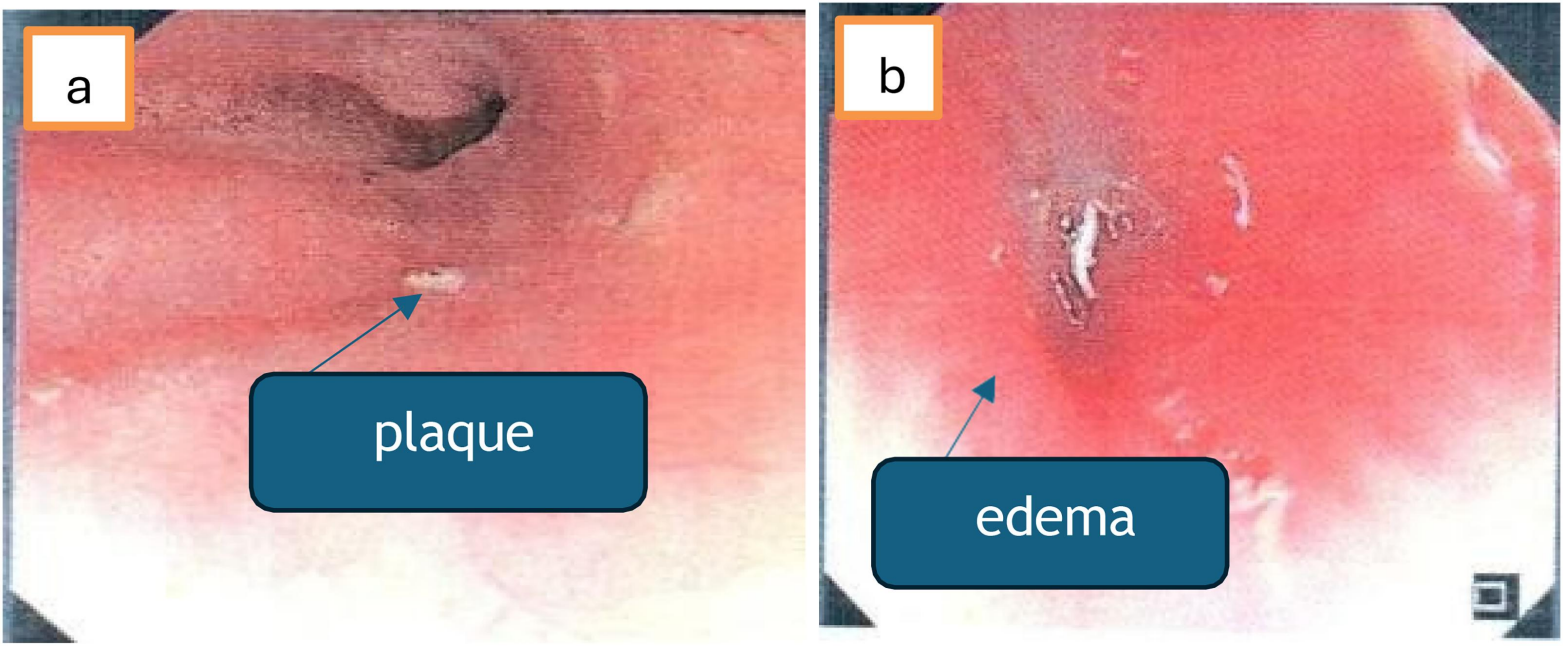
**(a)** Plaques and **(b)** edema.

OGD revealed white esophageal plaques (EREFS 1) (Figure 3).

*Mid esophagus:* spongiosis, no basal zone hyperplasia, severe inflammation with 10 eosinophils/HPF.
*Distal esophagus:* occasional eosinophils 0–2/HPF, no spongiosis.
*Gastric antrum and body:* mild gastric inflammation with 1 eosinophil/HPF

#### Treatment approach

Although the endoscopic findings did not meet the criteria for EoE, the clinical presentation displayed classic EoE signs, and the patient was treated empirically for suspected EoE. She was started on a PPI due to the severity of her symptoms while endoscopic evaluation was being planned. The eosinophil count was below the diagnostic threshold required for EoE, possibly due to early exposure to PPI therapy. Given the poor response to the PPI, she was subsequently treated with a 5-day course of oral prednisolone, followed by daily budesonide slurry therapy, in addition to wheat and dairy elimination.

#### Follow-up and outcome

After 1 month, she showed marked clinical improvement. A repeat esophagogastroduodenoscopy with biopsy was performed, demonstrating histologic remission. Repeat biopsies from both the upper and lower esophagus showed no spongiosis, no basal zone hyperplasia, and an eosinophil count of 0 eosinophils/HPF. On subsequent follow-up visits, abdominal pain had resolved, and the feeding tolerance had improved, indicating a good response to the combined pharmacologic and dietary therapy.

### Case report 4

#### Presenting symptoms

An adolescent boy in the age bracket of 14–18 years presented with a 1-year history of intermittent epigastric pain that worsened over 2 weeks, with nausea, vomiting, and bloating. Previously, he had been managed for constipation with a bowel washout followed by polyethylene glycol and sodium picosulfate, which provided partial relief. After 3 months, he still had intermittent pain and infrequent bowel movements.

#### Past medical history

He had been treated for *H. pylori* 3 years earlier and had allergic rhinitis managed with intranasal steroids. His dietary history revealed a high intake of wheat-based products. His family history was positive for atopy, with two siblings diagnosed with asthma.

Examination revealed palpable fecal masses and increased bowel sounds. Growth parameters were normal (53 kg, 1.65 m, BMI 19.5).

#### Endoscopic findings

**Figure 4:**
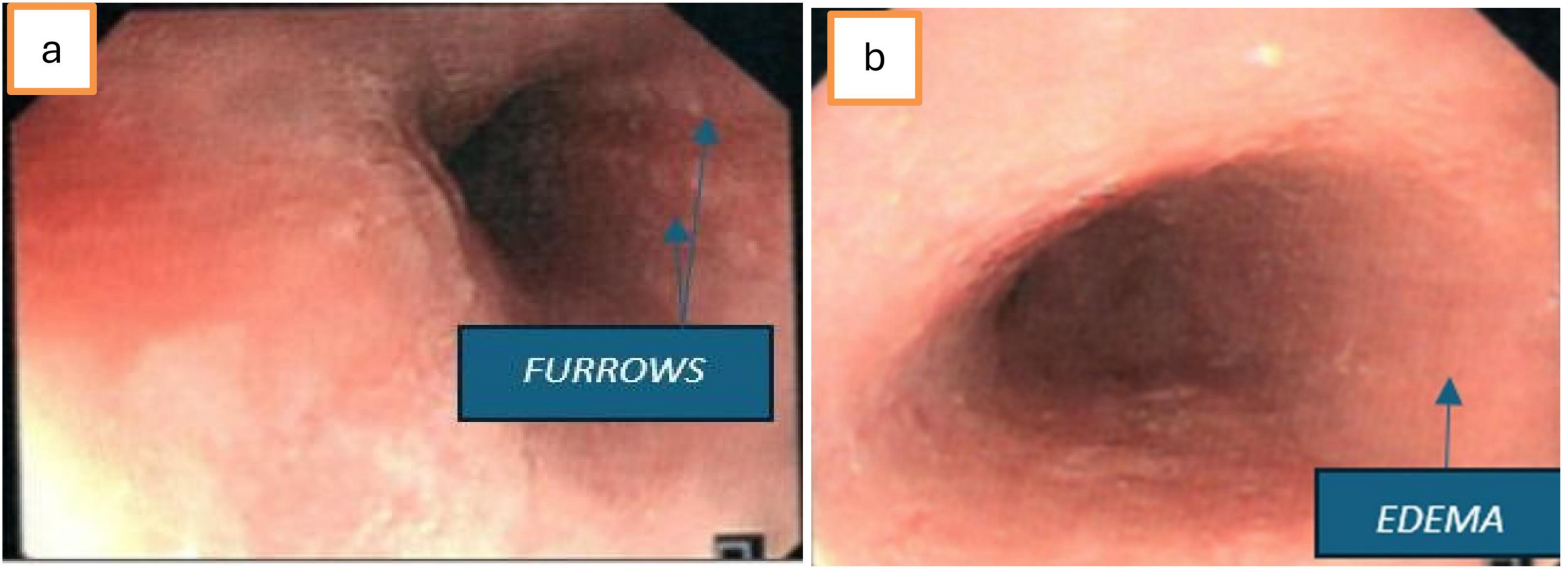
**(a)** Furrowing and **(b)** edema.

Upper endoscopy revealed white esophageal plaques, edema, exudates, and furrowing (EREFS 3) (Figure 4).

*Distal esophagus:* chronic inflammation with >20 eosinophils/HPF and marked basal zone hyperplasia.
*Gastric antrum and body*: mild active chronic inflammation with 3–4 eosinophils/HPF.

#### Treatment approach

The patient was diagnosed with confirmed EoE. He was started on sodium picosulfate, polyethylene glycol, sucralfate, and esomeprazole, along with the elimination of wheat and cow's milk.

#### Follow-up and outcome

One month later, he reported a marked improvement in pain, bloating, and bowel movement regularity. He continues follow-up in the pediatric gastroenterology clinic and remains clinically well. A repeat esophagogastroduodenoscopy with biopsy was not performed; therefore, histologic remission could not be assessed.

### Case report 5

#### Presenting symptoms

An adolescent boy in the age bracket of 9–13 years presented with a 1-year history of intermittent abdominal pain, nausea, and bloating.

Examination revealed constipation with palpable fecal masses. Following fecal disimpaction and maintenance laxatives, his bowel habits normalized, but his abdominal symptoms persisted.

#### Past medical history

The patient had a history of *H. pylori* infection, which had been treated 2 years earlier, and he consumed a wheat-rich diet. His family history was notable for atopic disease.

#### Endoscopic findings

**Figure 5:**
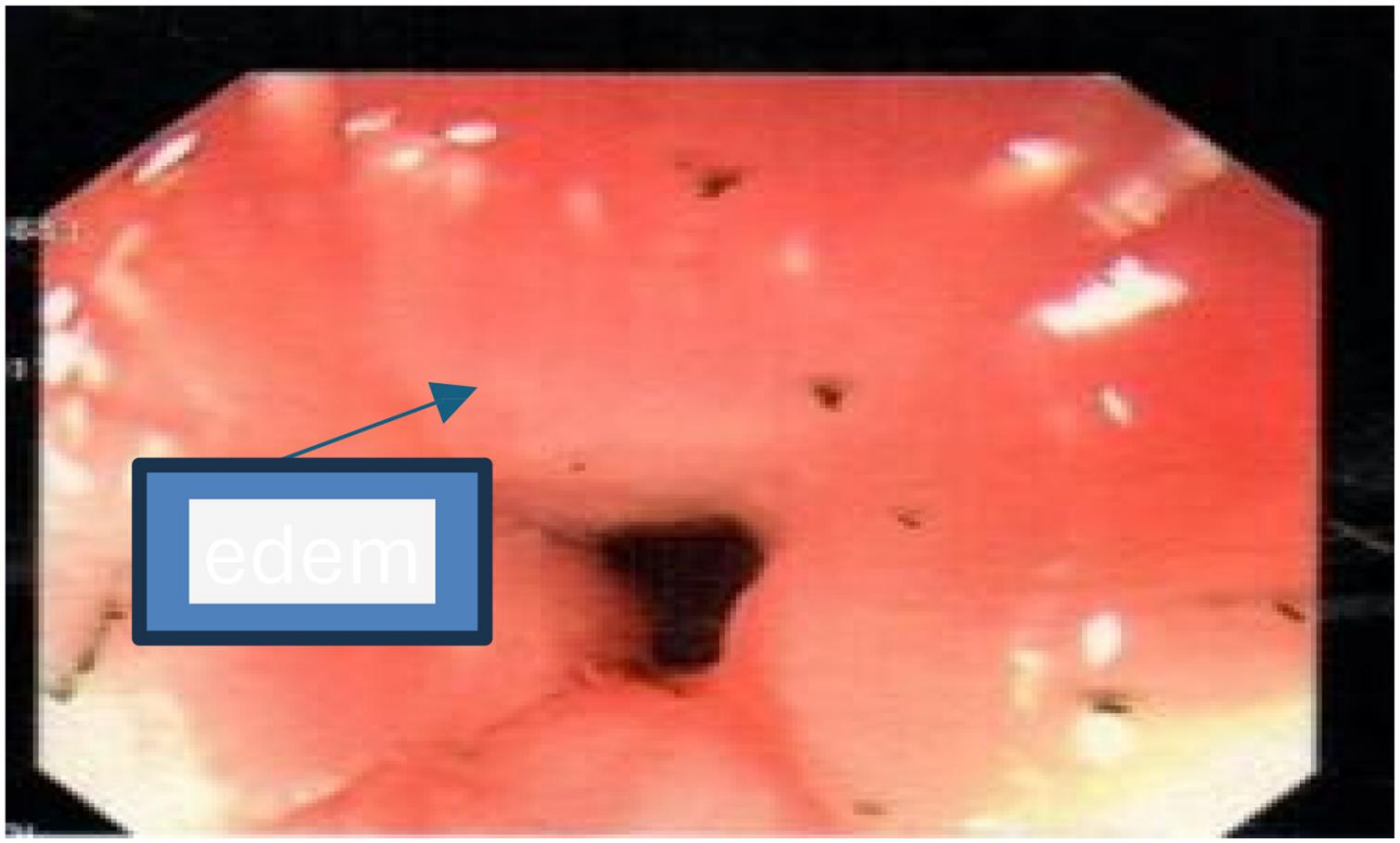
Endoscopic finding of esophageal edema for case 5.

Upper endoscopy demonstrated esophageal edema (EREFS 1) (Figure 5).

*Distal esophagus*: moderate active chronic inflammation with basal zone hyperplasia, spongiosis, and 20 eosinophils/HPF.
*Gastric antrum and body:* mild chronic inflammation with 4 eosinophils/HPF.

#### Treatment approach

The patient was diagnosed with confirmed EoE. He was started on esomeprazole, sucralfate, and dietary elimination of wheat and dairy.

#### Follow-up and outcome

At follow-up, he reported complete symptom resolution. At the time of reporting, a repeat endoscopy had not been performed; therefore, histologic remission could not be assessed.

### Case report 6

#### Presenting symptoms

A boy in the age bracket of 4–8 years with Klinefelter syndrome presented with persistent abdominal pain and poor appetite after transitioning from gastrostomy to oral feeding. He also experienced postprandial discomfort and occasional regurgitation.

#### Past medical history

Investigations done previously had shown severe gastroesophageal reflux and marked esophageal dysmotility. Treatment with domperidone and esomeprazole had provided partial relief.

#### Endoscopic findings

**Figure 6:**
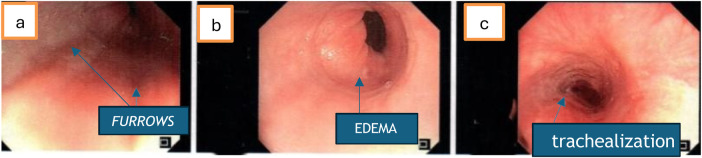
**(a)** Furrowing, **(b)** edema, and **(c)** trachealization for case 6.

Upper endoscopy revealed whitish nodules, mucosal furrowing, and trachealization (EREFS 4) (Figure 6).

*Mid esophagus*: spongiosis, basal zone hyperplasia, papillary elongation, and mild chronic inflammation with 10 eosinophils/HPF.
*Distal esophagus*: moderate chronic inflammation with spongiosis, basal zone hyperplasia, and papillary elongation with 14 eosinophils/HPF.
*Gastric antrum:* mild chronic inflammation with 5 eosinophils/HPF.

#### Other investigations

Complete blood counts were normal. Blood urea nitrogen was elevated at 9.2 mmol/L, with normal electrolytes and creatinine levels. C-reactive protein was slightly elevated at 11 mg/L. The serum-specific IgE food allergy test was positive for a hypersensitivity to cow's milk protein.

#### Treatment approach

The patient did not meet the criteria for confirmed EoE because his eosinophil count was <15/hpf. He was classified as having suspected EoE and was treated empirically. The patient was initiated on a strict elimination diet excluding cow's milk, with a plan to start budesonide slurry if his symptoms did not improve after the diet.

#### Follow-up and outcome

At follow-up, the patient reported improvement with elimination of cow's milk from his diet. He continues follow-up in the pediatric gastroenterology clinic and remains clinically well. A repeat esophagogastroduodenoscopy with biopsy was not performed; hence, histologic remission could not be assessed.

### Case report 7

#### Presenting symptoms

An obese adolescent girl in the age bracket of 14–18 years presented with chronic epigastric pain, initially treated as gastritis with lansoprazole and dietary modifications. Despite 2 months of therapy, the symptoms persisted, though gradual weight loss occurred.

#### Endoscopic findings

**Figure 7:**
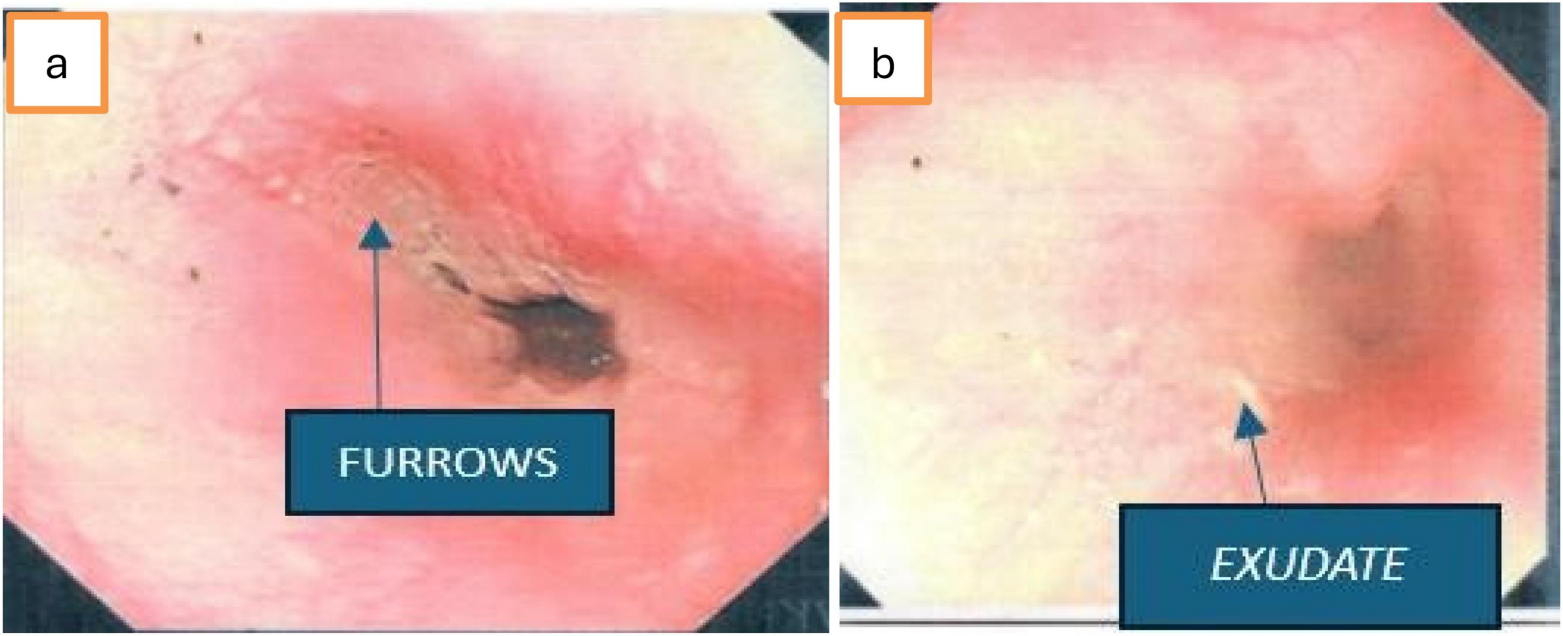
**(a)** Furrows and **(b)** exudates.

OGD revealed esophageal exudates, microabscesses, and furrowing (EREFS 3) (Figure 7).

*Mid esophagus*: no spongiosis or basal zone hyperplasia.
*Distal esophagus:* moderate active chronic inflammation with 40 eosinophils/HPF.
*Gastric body and antrum:* mild chronic inflammation with 1–2 eosinophils/HPF.

#### Other investigations

Complete blood count, liver function tests, and urea and electrolytes were within normal limits. The lipid profile showed borderline high cholesterol and borderline high low-density lipoprotein levels, with a high total cholesterol-to-high-density lipoprotein ratio.

#### Treatment approach

The patient was diagnosed with confirmed EoE and maintained on 12-hourly administration of lansoprazole.

#### Follow-up and outcome

While on treatment, moderate symptomatic improvement was noted. There was a reduction in epigastric pain and an improvement in overall gastrointestinal symptoms. A repeat esophagogastroduodenoscopy with biopsy had not been performed at the time of reporting; hence, histologic remission could not be assessed.

### Case report 8

#### Presenting symptoms

A boy in the age bracket of 9–13 years presented with a 1-year history of intermittent epigastric pain that had worsened over 1 week. His symptoms included postprandial, non-bilious vomiting, nausea, and malaise. The pain was burning, localized, food-aggravated, and relieved by vomiting. He had previously been empirically treated for *H. pylori* at another facility, with minimal improvement.

#### Endoscopic findings

**Figure 8:**
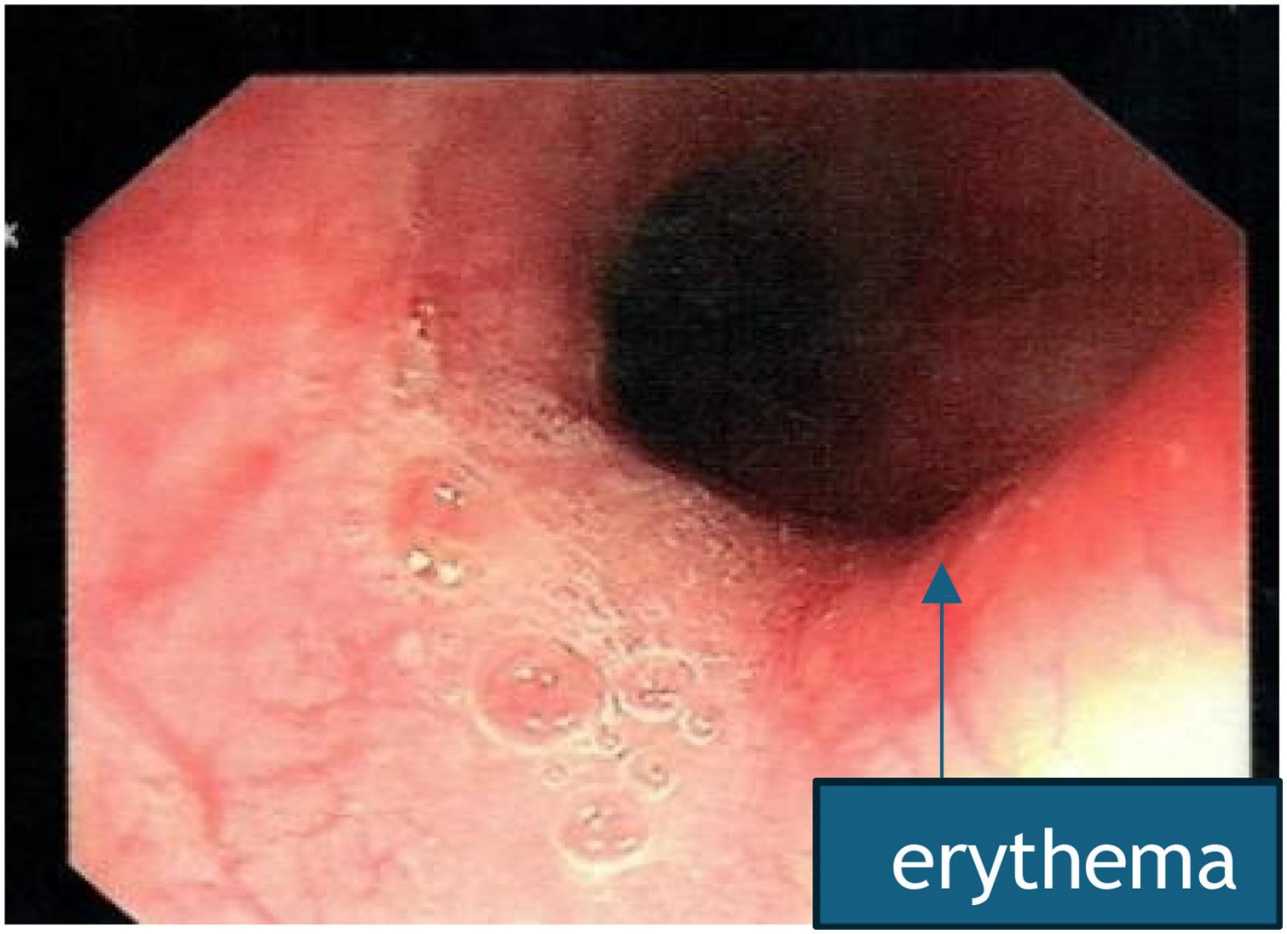
Distal esophageal erythema for case 8.

OGD revealed distal esophagitis (EREFS 0) (Figure 8).

*Distal esophagus:* moderate active chronic inflammation with 8–27 eosinophils/HPF, mild spongiosis, and basal zone hyperplasia.
*Gastric body and antrum*: mild chronic inflammation with 2–3 eosinophils/HPF.
*Colon:* mild active colitis with 5 eosinophils/HPF.

#### Other investigations

The liver function panel and lipase levels were all within normal limits.

#### Treatment approach

This patient was diagnosed with confirmed EoE based on the highest eosinophil count observed on biopsy. Upon admission, he received IV esomeprazole, ondansetron, sucralfate, fluids, and dietary advice. He was maintained on esomeprazole 20 mg twice daily, along with budesonide slurry.

#### Follow-up and outcome

On follow-up, symptoms were resolved, and the patient was weaned off therapy after 6 weeks. A repeat esophagogastroduodenoscopy with biopsy was performed 3 months after treatment, showing persistent distal esophagitis and nodular antral gastritis endoscopically. However, a repeat esophageal biopsy demonstrated histologic remission, with no intraepithelial eosinophils identified (0 eosinophils/HPF), no spongiosis, and no basal zone hyperplasia. Gastric biopsies showed mild chronic gastritis and were negative for *H. pylori*.

## Discussion

EoE is a chronic condition with a heterogeneous clinical presentation that varies with age, ranging from vomiting to long-term difficulty swallowing, with resultant growth impairment. A diagnosis is made based on symptoms of esophageal dysfunction in conjunction with histological evidence of a significant eosinophil count, provided that other causes of esophageal eosinophilia have been excluded (10, 11). As seen in case 3, the eosinophil count is below the threshold for EoE, which may be due to prior PPI exposure before the endoscopy, given the severity of the symptoms. EoE is more frequently reported in men and in individuals with a personal or family history of atopy; however, our case series was not designed to assess demographic patterns or risk factors (1, 2). While three of the patients in this series were boys and two had allergic rhinitis, these observations are purely descriptive and should not be interpreted as reflective of the population trend. Larger, systematically designed studies need to be conducted to assess the epidemiological statistics of EoE in our population.

Data on pediatric EoE in sub-Saharan Africa remain scarce, with the majority of existing literature originating from high-income countries. This case series aims to enhance regional knowledge by detailing the clinical presentation and management of EoE in our context. Notably, compared with reports from high-income countries, where early recognition is increasingly common, our patients appeared to have experienced symptoms for longer before diagnosis, suggesting possible delays in referral and limited access to specialized diagnostic services.

This underscores gaps in awareness and healthcare access that may affect disease recognition in our region (12).

GERD remains an important differential diagnosis for patients with EoE, posing significant diagnostic challenges. Emerging evidence suggests that EoE and GERD can coexist (10). In this case series, none of the patients had a formal diagnosis of GERD. However, three had a long-standing history of gastritis and demonstrated a suboptimal response to ideal proton pump inhibitor therapy. Given the small sample size and observational nature of this report, these findings underscore diagnostic complexity rather than establishing clinical associations.

OGD remains the gold-standard diagnostic tool, with the EREFS being a valuable and reliable scoring system for diagnosing EoE (13, 14). At least two biopsies from each level are required during OGD to improve diagnostic yield. However, allergy testing is not recommended unless deemed necessary (10, 15). According to the AGREE guidelines (16), patients with symptoms of EoE but with histological biopsies with eosinophils below the threshold of 15/hpf can be classified as suspected EoE and treated empirically.

Food allergy tests may be conducted as part of additional evaluations, but they are not mandatory. In cases 2 and 6, specific food IgE allergy tests returned positive results; however, these results primarily indicate sensitization rather than identifying the specific foods that cause esophageal inflammation. Therefore, it is crucial to emphasize that allergy testing should be interpreted as evidence of sensitization rather than conclusive proof of the foods that trigger the disease.

The principles of therapy focus on achieving histologic remission and restoration of normal growth and development in pediatric patients. Management requires a multidisciplinary approach at a tertiary level, involving pediatric gastroenterologists, nutritionists, pathologists, and, when necessary, allergists. Therapeutic options include dietary modifications and pharmacotherapy, such as acid-suppressing medications, topical corticosteroids, and dupilumab, with long-term follow-up being an essential component of the care plan (17).

Dietary therapy is an effective first-line treatment modality and supports the immune-mediated pathogenesis of EoE related to food antigens. In this series, wheat and dairy products were suspected triggers and patients showed noticeable clinical improvement following their elimination. However, dietary elimination should be undertaken cautiously and under the guidance of a qualified nutritionist to ensure nutritional adequacy and prevent compromise in growth. Established dietary approaches include the SFED, four-food and two-food elimination diets, elemental diet, and test-directed elimination diet. A meta-analysis demonstrated the varying remission rates across the various dietary strategies, with the elemental diet being the most effective, with a histologic remission rate of 90.8%, while SFED had a rate of 74% and the four-food elimination diet had a rate of 64% (18–20).

Low-potency corticosteroids such as budesonide or fluticasone, administered orally as a slurry, are generally well tolerated and effective in inducing remission (10). In this series, one patient achieved complete clinical and histological remission following a 10-week course of budesonide, while another demonstrated significant clinical improvement, including the ability to accept solid foods.

PPIs have also been used in the management of EoE, typically administered every 12 hours, with a systematic review and meta-analysis demonstrating a clinical response rate of 60.8% and a histologic remission rate of 50.5% (21).

Dupilumab, an IL-4 receptor alpha antagonist, inhibits IL-4 and IL-13 signaling pathways and abates the consequential inflammatory cascade mediated by Th2 cells. The FDA has approved its use for adults and for children over the age of one who weigh at least 15 kg. It is administered subcutaneously on a weight-based dosing regimen and is reserved for refractory EoE cases. However, its limited availability in our setting precluded its use in this series (10).

In conclusion, this case series highlights the upsurge of EoE in our setting and provides a descriptive overview of its clinical presentation and management. Further multicenter, prospective studies are needed to assess the prevalence, incidence, and effectiveness of the various treatment modalities in our context.

## Data Availability

The original contributions presented in this study are included in the article/Supplementary Material, further inquiries can be directed to the corresponding author.
